# Investigation of Polyhenolic Content of Rose Hip (*Rosa canina* L.) Tea Extracts: A Comparative Study

**DOI:** 10.3390/foods2010043

**Published:** 2013-02-05

**Authors:** Zeynep İlbay, Selin Şahin, Ş. İsmail Kırbaşlar

**Affiliations:** Department of Chemical Engineering, Engineering Faculty, Istanbul University, 34320 Avcılar, Istanbul, Turkey; E-Mails: zilbay@gmail.com (Z.İ.); krbaslar@istanbul.edu.tr (Ş.İ.K.)

**Keywords:** extraction effect, *Rosa canina* L., soxhlet method, total phenol content, ultrasound-assisted extraction

## Abstract

Three different brands of Rose hip (*Rosa canina* L.) tea were extracted with water, ethanol (EtOH), methanol (MeOH), and aqueous mixtures (50%, v/v) by ultrasound-assisted extraction (UAE) and Soxhlet methods. Total phenolic content was determined according to the Folin-Ciocalteu method. The results were presented by means of the extract yields and total phenolic contents, expressed in gallic acid equivalent (GAE) per g of dried matter (DM). The greatest amount of extract observed in tea samples was obtained by UAE through water with the value of 619.37 ± 0.58 mg/g DM. Regarding the phenolic content, the best result was achieved by the Soxhlet method through 50% MeOH mixture (59.69 ± 0.89 mg GAE/g DM), followed by the UAE method with water (48.59 ± 0.29 mg GAE/g DM).

## 1. Introduction

Oxidation is the most important process in aerobic life concerning energy production in the form of ATP. However, oxidation process in electron flow system may result in producing free radicals, known as reactive oxygen species (ROS). These ROS can cause membrane damage, protein modifications, and DNA damage [1]. Aerobic life has evolved antioxidant systems to suppress the unwanted effects of free radicals. Some of these antioxidants are produced in the body, and others are obtained through diet. Antioxidants, which are obtained through diet, are originated from natural and synthetic sources. The most common synthetic antioxidants, such as butlylatedhydroxyanisole (BHA) and butylatedhydroxytoluene (BHT), have been classified as primary antioxidants and have high manufacturing costs, so they are not economical. Furthermore, they are restricted for use in foods by legislative rules because of their carcinogenicity and toxicity [2,3,4]. Therefore, the interest of researchers tends to be toward finding new and safe antioxidants from natural sources, which are abound in various types of plant materials like herbs and spices [5,6]. Epidemiological studies revealed that numerous phytonutriens obtained from plant materials are beneficial in protecting human cells, serving as radical scavengers [7,8,9,10]. Vitamin A, E, C and phenolic compounds, including tannins, flavonoids, and lignins, in plants have antioxidant properties [11]. By adding these materials to fats, the lipid peroxidation process is retarded, thus the shelf life of food products is prolonged [12,13]. Furthermore, they play a crucial role by acting as scavengers of free radicals and lipid peroxidation inhibitors, and in the prevention of diseases caused by oxidative stress, such as cardiovascular disease, brain dysfunction, and cancer [14,15,16,17,18,19,20]. Therefore, human beings prefer to intake better quality food, which includes higher antioxidants in their diet.

*Rosa canina* L., known as rose hip, grows wildly in various regions of Turkey, is mostly used for the prevention and treatment of the common cold, gastrointestinal disorders, diabetes, kidney disorders, and other infections [21,22]. Due to its popularity as a medical remedy, *Rosa canina* L. has become a popular research subject for researchers, as well. Researchers have shown that the utilization of *Rosa canina* L. as a remedy in traditional folk medicine comes from its high content of phenolic compounds and minerals. In particular *Rosa canina* L. is a great source of ascorbic acids, tocopherols, bioflavonoids, tannins, pectin, minerals, aminoacids, flavonoids, unsaturated and polysaturated fatty acids, phospolipids, minerals, gallactolipids, and carotenoids [23]. 

In order to obtain target components in a plant matrix, an extraction method is applied as a unit operation to separate compounds from others [24]. Different kinds of solvents are used for the extraction of polyphenols such as ethanol (EtOH), methanol (MeOH), acetone, ethyl acetate, and aqueous mixtures of these solvents [25]. In order to obtain the highest amount of a desired component, not only the solvent-type but also extraction methods are relevant. Ultrasound-assisted extraction (UAE) is a novel extraction method, which is known as a green and innovative technique involving less time, energy, and solvent than traditional and conventional methods, such as soxhlet and maceration.

There are several studies on rose hip extraction with different solvent systems in the literature. Gao *et al.* [26] measured the antioxidant quantities in phenolic, lipophilic, and ascorbic fractions in samples, extracted from different dried and powdered rose hip species with EtOH. Daels-Rakotoarison *et al.* [27] investigated mainly phenolic-materials extracted from crushed rose hip with acetone/water using the maceration method. Lattanzio *et al.* [28] studied antioxidant activity of *Rosa canina* L. extracts obtained using ethanol/water solution with the maceration method. Wenzig *et al.* [29] examined total phenolic, ascorbic acid, and antioxidant activity of *Rosa canina* extracts by applying the Soxhlet method with *n*-hexane, dichloromethane, and MeOH. The aim of the present research is to investigate the obtaining methods with the highest yield of extract, rich in polyphenols, of the three different commercial brands of rose hip tea.

## 2. Experimental Section

### 2.1. Materials

#### 2.1.1. Plant Material

Three different commercial brands of rosehip tea were purchased from a local market in Istanbul, Turkey. They were individually blended and stored at ambient temperature, in the dark, until use.

#### 2.1.2. Chemicals and Reagents

Ethanol and methanol were provided by Merck and were of >99.5% and >99.8% mass fraction purity, respectively. Folin-Ciocalteu reagent, sodium carbonate, and gallic acid were purchased from Sigma-Aldrich, Steinheim, Germany. Eighteen milliomega deionized water from a Millipore Milli-Q water purification system was used to prepare mixtures analyses. 

### 2.2. Methods

#### 2.2.1. Ultrasound-Assisted Extraction

Ultrasound-assisted extraction was conducted in an ultrasonic bath (Protech, İstanbul, Turkey) with a frequency of 40 kHz, at 25 °C. Dried and ground plants and 10 mL of solvent were sealed in an Erlenmayer flask and placed into the bath. The mixture was centrifuged (CN 180, Nüve, Ankara, Turkey) at 5000× *g* for 25 min. After centrifugation, the supernatant was filtered through a 0.45 µm syringe filter and stored at −80 °C until analysis for the biochemical measurements. For the extract yields, the solvent was removed from a certain quantity of extract in a rotary evaporator (Buchi, Flawil, Switzerland).

#### 2.2.2. Soxhlet Method

Ten grams of dried and ground plants were placed in a Soxhlet apparatus and extracted with 250 mL of solvent in a volumetric flask containing glass beads for 24 h. After extraction, the extract solution was filtered through a 0.45 µm syringe filter and stored at −80 °C until analysis of the biochemical measurements. For the extract yields, the solvent was removed from the extract in a rotary evaporator (Buchi, Flawil, Switzerland).

### 2.3. Total Phenols Determination

The concentration of the total polyphenols in extracts was measured by UV-spectrophotometry (Optima SP-300, Tokyo, Japan), based on a colorimetric oxidation/reduction reaction. The total phenolic content was determined according to the Folin-Ciocalteu method by the following procedure of Malik and Bradford [30]. Folin-Ciocalteu reagent was used as oxidizing agent. To 10 µL Folin-Ciocalteu reagent of extract, 190 µL of water was added. One milliliter of Folin-Ciocalteu reagent, and 800 µL of Na_2_CO_3_ (75%, w/v) were added. The samples were incubated for 30 min. The absorbance was measured at 760 nm. The amount of total phenolic content was expressed in gallic acid equivalent per g of dried leaf (mg GAE/g dried matter). Calibration curves were calculated using pure gallic acid with different concentrations for each solvent system.

### 2.4. Statistical Analysis

Three replicate extractions were carried out for each of the samples followed by a minimum of three spectrophotometric measurements from each extract. Statistical analysis on the means of triplicate experiments was carried out using the ANOVA procedure of the InStat^®^ software, version 3.0 (GraphPad, San Diego, CA, USA). Tukey’s test of significance between means was used for illustration of significance

## 3. Results and Discussion

### 3.1. Influence of Solvent Type on UAE Efficiency Depending on the Tea Brand

Table 1 presents the results, accounting for the effect of solvent type (EtOH, MeOH, water, and the aqueous mixtures of those solvents) on the extract yield and total phenolic content of each brand of rose hip tea (A, B, C) through UAE as a function of time. Both the extract yield and TPC of all solvent extracts increased steadily as a function of time.

**Table 1:** Extract yield and total phenolic content of extracts obtained by ultrasound-assisted extraction (UAE) at different periods through various solvent percentages depending on the tea brand^ x^.

Solvent type	Solvent Percentage (% (v/v))	Tea brand	Time (min)	Extract yield^ y^ (mg/g DM)	Total phenolic content^ z^ (mg GAE/g DM)
EtOH	100	A	30	37.56 ± 0.51	2.25 ± 0.05 a
			60	63.86 ± 0.50	3.45 ± 0.16 b
			90	71.00 ± 0.73	6.52 ± 0.26 c
		B	30	76.54 ± 0.54	2.95 ± 0.07 ab
			60	99.82 ± 0.42	3.58 ± 0.15 bd
			90	105.33 ± 0.51	4.30 ± 0.13 de
		C	30	84.22 ± 0.32	7.54 ± 0.21 c
			60	92.63 ± 0.46	4.38 ± 0.04 de
			90	114.20 ± 0.25	4.69 ± 0.06 e
MeOH	100	A	30	273.13 ± 0.79	11.86 ± 0.21 f
			60	295.08 ± 0.54 a	16.15 ± 0.24
			90	333.13 ± 0.64 b	18.36 ± 0.29
		B	30	293.65 ± 0.43 a	12.06 ± 0.19 f
			60	325.70 ± 0.60 c	12.33 ± 0.16 fg
			90	327.08 ± 0.64 c	13.42 ± 0.22 g
		C	30	271.15 ± 0.43	11.91 ± 0.30 f
			60	308.03 ± 0.71	12.52 ± 0.21 fg
			90	333.80 ± 0.95 b	13.26 ± 0.23 g
EtOH	50	A	30	455.22 ± 0.61	21.58 ± 0.27 h
			60	500.38 ± 0.64	20.23 ± 0.46 i
			90	524.45 ± 0.62 d	31.37 ± 0.49
		B	30	407.98 ± 0.62 e	20.28 ± 0.14 i
			60	523.88 ± 0.53 d	21.70 ± 0.12 h
			90	578.96 ± 0.63	29.80 ± 0.19 j
		C	30	337.13 ± 0.78	26.26 ± 0.41
			60	457.79 ± 0.80	29.88 ± 0.47 j
			90	463.07 ± 0.63	27.98 ± 0.18
MeOH	50	A	30	492.83 ± 0.84 f	41.57 ± 0.30 l
			60	527.49 ± 0.78	48.42 ± 0.50 k
			90	542.81 ± 0.80	49.26 ± 0.52 k
		B	30	492.57 ± 0.42 f	43.25 ± 0.29 m
			60	532.30 ± 0.89	42.12 ± 0.30 l
			90	617.01 ± 0.45	45.66 ± 0.23 n
		C	30	409.44 ± 0.39 e	40.11 ± 0.31
			60	436.55 ± 0.35	42.05 ± 0.49 lo
			90	467.49 ± 0.43	44.08 ± 0.41 m
Water	100	A	30	404.23 ± 0.61	45.42 ± 0.33 n
			60	476.44 ± 0.64	47.34 ± 0.47 k
			90	548.76 ± 0.71	54.85 ± 0.59
		B	30	537.91 ± 0.36	44.28 ± 0.21 m
			60	569.95 ± 0.67	43.75 ± 0.18 m
			90	619.37 ± 0.58	47.91 ± 0.34 k
		C	30	363.53 ± 0.37	42.39 ± 0.40 l m
			60	429.51 ± 0.62	42.68 ± 0.35 mo
			90	484.85 ± 0.59	48.59 ± 0.29 k

^x^ Means within the same column sharing a common letter indicate nonsignificance at *p* > 0.05; ^y^ Data are expressed as the mean (*n* = 3) ± S.D.; ^z^ Data are expressed as the mean (*n* = 9) ± S.D.

The mechanism of the UAE process here has two main stages. First, dissolution of soluble components on surfaces of the plant matrix occurs, which is also called “washing”. Secondly, mass transfer of the solute from the plant matrix into the solvent by diffusion and osmotic processes, which is known as “slow extraction” [31,32,33,34]. Washing occurs at the beginning of the extraction with a rapid increase. Generally, after 60 min, the slow extraction is observed by a low raise in both concentrations. Therefore, 90 min is accepted as the optimum time in this process, giving the equilibrium concentration of the extracted leaves (Table 1).

The extract yield of UAE ranged from 37.56 to 619.37 mg/g DM through various solvent systems. While pure water as extraction solvent was distinguished by the highest level of extract yield, pure ethanol showed the poorest level of all the other solvent systems. With respect to influence of the solvent ratio, there was a high recovery of extract at high water quantities. As the alcohol content decreased, there was a rise in the recovery of the extract for both EtOH and MeOH mixtures. In regards to total phenolic content, the quantity changed between 2.25 and 54.85 mg GAE/g DM. The results clearly showed that extract obtained by pure water had the highest total phenolic content, followed by aqueous MeOH extract. In addition, there was no significant difference (*p* > 0.05) between the values of phenolic content obtained by pure water and that of 50% MeOH solution (48.42, 49.26, 47.34, 47.91 and 48.59 mg GAE/g DM, respectively). Sultana *et al.* [35] also showed similar results to this study with different plant specimens.

Fifty percent alcoholic solutions showed much better performance than the pure alcohols, which is a result of water’s altering of the plant structure by swelling the matrix, enabling the solvent to more completely penetrate the plants. Accordingly, water is acting as the plant-swelling agent, while alcohol is believed to disrupt the bonding between the solutes and plant matrices [36,37]. Another explanation might be the high dielectric constant of water, which leads to an increase of polarity indices of alcohol with its water solution [38].

### 3.2. Influence of Solvent Type on Soxhlet Method Efficiency Depending on the Tea Brand

Table 2 indicates the influence of solvent type on the extract yield and total phenolic content of each rose hip tea extract obtained through the Soxhlet method. Selecting the solvent type is of great importance to extracting target compounds from plant material [39,40]. A polar solvent is necessary to be able to extract polar phenolic compounds of plant extract [41,42]. The extract obtained by pure water exhibited the highest yield with the value of 566.02 mg/g DM, whereas the lowest total phenolic was extracted by 50% EtOH solution, with the quantity of 153.35 mg/g DM. Daels-Rokotoparison *et al.* [27] investigated the extraction of rose hip, from France, using maceration at 40 °C in acetone/water solution, and achieved 197.24 mg extract per 100 g of dried rose hip.

**Table 2:** Extract yield and total phenolic content of extracts obtained by the Soxhlet method through various solvent percentages, depending on the tea brand ^x^.

Solvent type	Solvent Percentage (% (v/v))	Tea brand	Extract yield^ y^ (mg/g DM)	Total phenolic content^ z^ (mg GAE/g DM)
EtOH	100	A	213.34 ± 0.57	16.63 ± 0.30 a
		B	187.31 ± 0.43	14.46 ± 0.27 b
		C	202.42 ± 0.52	15.01 ± 0.22 b
MeOH	100	A	446.84 ± 0.62	27.08 ± 0.43
		B	551.44 ± 0.69	24.63 ± 0.33 c
		C	486.13 ± 0.83	23.29 ± 0.26 c
EtOH	50	A	153.35 ± 0.26	51.18 ± 0.81
		B	159.82 ± 0.33 a	43.83 ± 0.58
		C	161.19 ± 0.35 a	41.52 ± 0.30
MeOH	50	A	407.15 ± 0.98	59.69 ± 0.89
		B	350.16 ± 0.48	57.26 ± 0.83
		C	328.74 ± 0.63	48.69 ± 0.53
Water	100	A	566.02 ± 0.87	18.07 ± 0.39 a
		B	531.76 ± 0.77	13.24 ± 0.23 b
		C	462.55 ± 0.82	15.74 ± 0.14 ab

^x^ Means within the same column sharing a common letter indicate nonsignificance at *p* > 0.05; ^y^ Data are expressed as the mean (*n* = 3) ± S.D.; ^z^ Data are expressed as the mean (*n* = 9) ± S.D.

The extracts obtained by pure water and EtOH shared the poorest yield, with the values of 13.24 and 14.46 mg GAE/g DM, which are not significantly different at *p* > 0.05 (Table 2). The maximum total phenolic was extracted by 50% MeOH solution, with the quantity of 59.69 mg GAE per g of dried matter, showing approximately 4.5 times better performance than pure water and EtOH in terms of phenolic quantity. Although the extract yield obtained by water was the highest of all solvent systems, the MeOH mixture was more efficient, with respect to phenolic content, than that of water. This can be explained by the long overheating effect of the higher boiling temperature of water, which leads to phenol degradation through the Soxhlet extraction method. Gao *et al.* [26] studied rose hip fruits from Santiago, Chile using 50% ethanol solution, under shaking at 40 °C for 24 h. The total phenolic yield was found out as 62.79 mg GAE/g DM.

Szentmihályi *et al.* [43] investigated the extraction efficiencies of the UAE and Soxhlet methods using hexane as solvents with waste hip seeds from *Rosa canina* L. Their results showed the same tendency as those of the present study. 

## 4. Conclusions

The differences in the extract and phenolic yields of three tea brands (A, B, and C) might be attributed to several agronomical and technological factors such as harvesting period, plant age, degree of ripeness, geographical origin, cultivar, phonological stage during sampling, moisture content, degree of contamination of soil, and industrial processes employed for grinding and storage, regardless of extraction method and solvent type. 

Considering the health safety properties, UAE with pure water was found to be an efficient method to extract the polyphenols present in the plant teas. 

Addition of water to alcohol improved the extraction of total phenolics, which is the result of water’s swelling effect on the plant matrix.

It is generally known that high yield of extract is achieved by using the Soxhlet method, which is a result of higher operating temperature as well as longer extraction time and high solvent/plant material. The viscosity and density of the solvent decreases, leading to fast mass transfer at high temperature.

With respect to extraction methods, although high yields were achieved by the Soxhlet method, a general comparison between the Soxhlet method and UAE cannot be established, where UAE considers short processing time and low solvent consumption. The Soxhlet method could be disadvantageous from the point of product quality leading to target compounds with unpleasant aromas because of the long extraction time and high temperatures.

As far as this research is concerned, it is recommended that rose hip tea extracts should be investigated more comprehensively in terms of individual components involved in its structure for a potential source of food additives or supplements.
